# Impact on grafted kidney function of rocuronium-sugammadex vs cisatracurium-neostigmine strategy for neuromuscular block management. An Italian single-center, 2014-2017 retrospective cohort case-control study

**DOI:** 10.1186/s13741-021-00231-2

**Published:** 2022-01-13

**Authors:** M. Carron, G. Andreatta, E. Pesenti, A. De Cassai, P. Feltracco, F. Linassi, M. Sergi, C. Di Bella, M. Di Bello, F. Neri, C. Silvestre, L. Furian, P. Navalesi

**Affiliations:** 1 Department of Medicine, DIMED, Section of Anesthesiology and Intensive Care, University of Padova, Via V. Gallucci, 13, 35121 Padova, Italy; 2 Institute of Anesthesia and Intensive Care, Azienda Ospedale Università Padova, Via Giustiniani 2, 35128 Padova, Italy; 3 Department of Anesthesia and Intensive Care, Ca’ Foncello Treviso Regional Hospital, Piazzale Ospedale 1, 31100 Treviso, Italy; 4 Department of Surgical, Oncological and Gastroenterological Sciences, Kidney and Pancreas Transplantation Unit, University of Padova, Via Giustiniani 2, 35128 Padova, Italy; 5 Kidney and Pancreas Transplantation Unit, Azienda Ospedale Università Padova, Via Giustiniani 2, 35128 Padova, Italy

**Keywords:** Anesthesia, Neuromuscular block, Rocuronium, Cisatracurium, Sugammadex, Neostigmine, End-stage renal disease, Kidney transplantation

## Abstract

**Background:**

The impact of sugammadex in patients with end-stage renal disease undergoing kidney transplantation is still far from being defined. The aim of the study is to compare sugammadex to neostigmine for reversal of rocuronium- and cisatracurium-induced neuromuscular block (NMB), respectively, in patients undergoing kidney transplantation.

**Methods:**

A single-center, 2014-2017 retrospective cohort case-control study was performed. A total of 350 patients undergoing kidney transplantation, equally divided between a sugammadex group (175 patients) and a neostigmine group (175 patients), were considered. Postoperative kidney function, evaluated by monitoring of serum creatinine and urea and estimated glomerular filtration rate (eGFR), was the endpoint. Other endpoints were anesthetic and surgical times, post-anesthesia care unit length of stay, postoperative intensive care unit admission, and recurrent NMB or complications.

**Results:**

No significant differences in patient or, with the exception of drugs involved in NMB management, anesthetic, and surgical characteristics, were observed between the two groups. Serum creatinine (median [interquartile range]: 596.0 [478.0-749.0] vs 639.0 [527.7-870.0] μmol/L, *p* = 0.0128) and serum urea (14.9 [10.8-21.6] vs 17.1 [13.1-22.0] mmol/L, *p* = 0.0486) were lower, while eGFR (8.0 [6.0-11.0] vs 8.0 [6.0-10.0], *p* = 0.0473) was higher in the sugammadex group than in the neostigmine group after surgery. The sugammadex group showed significantly lower incidence of postoperative severe hypoxemia (0.6% vs 6.3%, *p* = 0.006), shorter PACU stay (70 [60-90] min vs 90 [60-105] min, *p* < 0.001), and reduced ICU admissions (0.6% vs 8.0%, *p* = 0.001).

**Conclusions:**

Compared to cisatracurium-neostigmine, the rocuronium-sugammadex strategy for reversal of NMB showed a better recovery profile in patients undergoing kidney transplantation.

## Introduction

Kidney transplantation represents the best option to improve survival and quality of life in patients with end-stage renal disease (ESRD) (Kellar, 2015).

The surgical procedure of kidney transplantation is generally performed under general anesthesia and presents significant challenges for the anesthesiologist (Martinez et al. 2013). A careful anesthetic approach is highly recommended to improve outcomes (Martinez et al. 2013; De Gasperi et al. 2014; Mittel and Wagener, 2017; Wagener et al. 2020). The management of neuromuscular block (NMB) deserves particular attention (Martinez et al. 2013; Mittel and Wagener, 2017) in order to reduce the incidence of complications due to postoperative residual NMB (De Gasperi et al. 2014; Miskovic and Lumb, 2017). Patients receiving, compared to those not receiving, neuromuscular blocking agents (NMBAs) during general anesthesia are at significantly increased risk of postoperative respiratory complications (adjusted odds ratio [aOR]: 1.86) (Kirmeier et al. 2019). Benzylisoquinolinium compounds, such as cisatracurium, and aminosteroid NMBAs, such as rocuronium, are commonly used during general anesthesia for kidney transplantation. There is no evidence supporting the superiority of a specific NMBA. Cisatracurium seems to benefit from certain favor among anesthesiologists because it is inactivated by Hofmann elimination and hydrolysis by esterases independent of renal function, whereas rocuronium is characterized by organ-independent elimination. However, both are associated with slightly prolonged duration of action and require careful neuromuscular function monitoring for safe recovery at the end of surgery (Della Rocca et al. 2003; Martinez et al. 2013; Mittel and Wagener, 2017). While proper neuromuscular function monitoring is crucial in avoiding postoperative complications, particularly respiratory complications (Blobner et al. 2020), the choice of reversal drug seems to be no less important (Kheterpal et al. 2020). Compared to neostigmine, an acetylcholinesterase inhibitor traditionally used for reversal of NMB, the use of sugammadex, a modified γ-cyclodextrin developed for the reversal of NMB induced by aminosteroid NMBAs, particularly rocuronium, was associated with a significantly lower incidence of major pulmonary complications (Kheterpal et al. 2020).

Sugammadex is a highly hydrophilic drug and acts in the plasma by encapsulating and inactivating unbound rocuronium to form a 1:1 water-soluble complex. Urinary excretion is the main route of elimination of sugammadex and the sugammadex-rocuronium complex. At this time, sugammadex administration is not recommended by the manufacturer for subjects with severe renal impairment (creatinine clearance [CrCl] < 30 mL/min), including those undergoing standard forms of dialysis (EMA 2021).

The safety profile of sugammadex observed in clinical studies involving subjects with severe renal impairment (Staals et al. 2008; de Souza et al. 2015) has encouraged its use in clinical practice in patients with ESRD (Adams et al. 2020; Paredes et al., 2020), particularly in those undergoing kidney transplant (Unterbuchner, 2016; Ono et al. 2018; Arslantas and Cevik, 2019; Adams et al. 2020; Vargas et al. 2020). However, only retrospective analyses including reports (Unterbuchner, 2016) or small cohorts of patients are available in the literature (Ono et al. 2018; Arslantas and Cevik, 2019; Adams et al. 2020; Vargas et al. 2020). Furthermore, no data exist on the use of sugammadex administered for reversal of deep NMB in patients undergoing kidney transplantation.

So, the aim of our study is to evaluate the impact of sugammadex, given at recommended doses for reversal of a moderate or deep rocuronium-induced NMB, compared to neostigmine, administered for reversal of moderate cisatracurium-induced NMB, on renal function in a large cohort of patients undergoing kidney transplantation.

## Materials and methods

### Ethical statement and study approval

All procedures performed in the study were in accordance with the ethical standards of the institutional and/or national research committee and with the 1964 Helsinki Declaration and its later amendments or comparable ethical standards. STROBE recommendations for cohort case-control studies were followed.

This retrospective observational study was approved by our Institutional Review Board (Ethics Committee in Clinical Research—CESC of Padova, Italy, prot.n.42587, 16 July 2020), which waived the requirement to obtain patients’ written informed consent (the data were analyzed retrospectively and anonymously).

### Patients

A total of 350 patients with ESRD undergoing kidney transplantation at our hospital were evaluated. Patients were recruited consecutively until the sample size was achieved.

The anesthesia and medical records and the information system’s computer database were used to retrieve data about all patients (age ≥ 18 years) with ESRD who received sugammadex or neostigmine to reverse rocuronium- or cisatracurium-induced NMB, respectively, during inhalational or intravenous anesthesia for kidney transplantation. Each anesthesia and medical record was reviewed for preoperative, intraoperative, and postoperative data up to 5 days after surgery. Patient demographics, comorbidities (e.g., history of neurological, respiratory, cardiac, abdominal, and metabolic disease), perioperative data including kidney function (serum creatinine and urea, estimated glomerular filtration rate [eGFR]), and postoperative events were considered.

Two distinct time periods that define the matched exposure groups were considered: the pre-sugammadex period, 2014-2015 (from which cisatracurium-neostigmine-treated patients were identified), and the sugammadex period, 2016-2017 (from which rocuronium-sugammadex-treated patients were identified). Sugammadex was introduced in Padua University Hospital in January 2013 and was initially restricted to emergency reversal and routine reversal of rocuronium-induced NMB in selected high-risk patients undergoing anesthesia (Carron M, Baratto F 2016). In January 2016, sugammadex use was allowed for routine reversal. This resulted in a switch from utilization of the cisatracurium-neostigmine to the rocuronium-sugammadex strategy. Neostigmine was administered to reverse only moderate cisatracurium-induced NMB, while sugammadex was used for both deep and moderate rocuronium-induced NMB at the end of surgery.

Standard monitoring was adopted, including deep anesthesia and neuromuscular function monitoring. A train-of-four ratio (TOFR) ≥ 0.90 was adopted as the criterion for tracheal extubation (Brull and Kopman, 2017). All patients received antibiotic prophylaxis (piperacillin 2 g) before surgery, immunosuppression (thymoglobulin 1-1.5 mg/kg or basiliximab 20 mg, and methylprednisolone 500 mg) at the start of surgical procedure, and diuretics (furosemide 100 mg and mannitol 18% 80 mL) during surgery after anastomosis of the renal artery.

After surgery, patients were transferred to the post-anesthesia care unit (PACU). Level of consciousness, respiratory rate, pulse oximetry, heart rate, and arterial blood pressure were monitored until discharge to the surgical ward. Pain and postoperative nausea and vomiting (PONV) were assessed using a numeric rating scale (NRS) from 0 = no pain or nausea to 10 = worst possible pain or nausea. Patients were also assessed for clinical evidence of residual or recurrent NMB (e.g., muscle weakness, oxygen desaturation, hypoventilation, critical respiratory event). Patients with a pain NRS score of > 3 in the PACU received rescue analgesics (paracetamol 1 g and tramadol 1 mg/kg intravenously). Patients with a PONV NRS score of > 3 received a rescue dose of droperidol 0.625-1.25 mg intravenously.

### Endpoints

Serum creatinine (primary endpoint) and serum urea and eGFR (secondary endpoints) for monitoring kidney function for up to 5 days after surgery represented the main endpoints of the study. Other endpoints were anesthetic and surgical times, length of PACU stay, intensive care unit (ICU) admission, clinical evidence of postoperative respiratory complications (e.g., hypoxemia with peripheral arterial blood oxygen saturation [SaO_2_] < 90%, critical respiratory event) or cardiovascular event (e.g., stroke, myocardial ischemia, heart failure, hypertension, arrhythmia), PONV NRS score of > 3, pain NRS score of > 3, residual or recurrent NMB, and presence of any other postoperative complications within 24 h after surgery that required treatment.

For respiratory function, gas exchange analysis of arterial blood (pH, arterial partial pressures of oxygen [PaO_2_] and carbon dioxide [PaCO_2_]) performed 15-20 min after tracheal extubation was considered. For cardiac function, heart rate (HR) and systolic (SBP) and diastolic (DBP) arterial blood pressures evaluated 15-20 min after tracheal extubation were considered.

Data were collected by researchers without any involvement in the management of patients. They created a dataset with anonymized data for statistical analysis performed by researchers not involved with data collection.

### Statistical analysis

The sample size was based on the following assumptions: a mean difference of 44.2 μmol/L of serum creatinine in the first postoperative day between the sugammadex group and the neostigmine group as clinically relevant in the postoperative period (Kork et al. 2015; Gameiro et al. 2018), type I error equal to 0.05, and type II error equal to 0.2 (power [1−β] = 0.8). Considering these assumptions, the sample size was calculated as 350 patients, equally divided between the sugammadex group (175 patients) and the neostigmine group (175 patients).

Descriptive analysis was used to summarize the sample’s characteristics. The normality of the distribution of quantitative characteristics was analyzed using the Shapiro-Wilk test. Continuous normally distributed variables are expressed as mean ± standard deviation (SD). Median and interquartile range (IQR) values are reported for non-normally distributed variables. The two-tailed Student’s t test or two-tailed Mann-Whitney *U* test was used to compare normally and non-normally distributed variables, respectively, between the sugammadex and neostigmine groups. Categorical data were reported as an absolute number and as a percentage (%) and compared using a *χ*^2^ or Fisher’s exact test. To determine the strength and direction of association between two variables, Bravais-Pearson’s correlation test was used for normally distributed variables, and Spearman’s rank correlation test was used for non-normally distributed variables. Multiple linear regression analysis was used to examine the relationship between one dependent variable and the independent variables. Using the Akaike information criterion, backward and forward stepwise regression was performed to select the best model. Correlation coefficients (CCs), estimate coefficients (ECs), standard errors (SEs), *t* values, and *p* values were determined. Statistical significance was set at *p* values < 0.05. All statistical analyses were performed using R version 3.4.0 (2017-04-21).

## Results

No significant differences in demographic or other patient characteristics were observed between the sugammadex and neostigmine groups (Table 1). With the exception of NMBAs and reversal drugs, no differences in anesthetic and surgical characteristics were observed between the two groups (Table 2). Sugammadex was administered for reversal of moderate and deep NMB in 57.7% and 42.3% of cases, respectively. In the postoperative period, the sugammadex group showed significantly lower incidence of hypoxemia, shorter PACU stays, and reduced ICU admissions (Table 2). No patient in either group exhibited clinical evidence of major postoperative complications.

**Table 1:** Patients’ characteristics

Characteristic	NEO (***n*** = 175)	SUG (***n*** = 175)	***P*** value
Sex, M/F, *n* (%)	117 (66.9)/58(33.1)	104 (59.4)/71 (40.6)	0.184
Age, years [IQR]	52 [40.5-63]	52 [43.5-60]	0.654
Height, cm [IQR]	170 [165-176]	170 [164-176]	0.355
Weight, kg [IQR]	70 [62-80]	68 [55-78]	0.186
BMI, kg/m^2^ [IQR]	24.22 [22-26.2]	23.6 [20.4-26.1]	0.194
ASA, II/III/IV, *n* (%)	34 (19.4)/137(78.3)/4 (2.3)	26 (14.9)/148(84.6)/1 (0.6)	0.197
Neurovascular disease, *n* (%)	6 (3.4)	10 (5.7)	0.444
Respiratory disease, *n* (%)	18 (10.3)	26 (14.9)	0.195
• Obstructive, *n* (%)	8 (4.6)	11 (6.3)	0.638
• Other, *n* (%)	10 (5.7)	15 (8.6)	0.407
Cardiovascular disease, *n* (%)	137 (78.3)	147 (84)	0.219
• Hypertension, *n* (%)	124 (70.9)	130 (74.3)	0.549
• Other, *n* (%)	13 (7.9)	17 (9.7)	0.529
Abdominal disease, *n* (%)	63 (36)	79 (45.1)	0.102
Impaired glucose tolerance, *n* (%)	25 (14.3)	30 (17.1)	0.557
Dyslipidaemia, *n* (%)	30 (17.1)	32 (18.3)	0.889
Other disease, *n* (%)	16 (9.1)	13 (7.4)	0.699
Previous KTx, *n* (%)	18 (10.3)	16 (9.1)	0.857

*NMB* neuromuscular block, *NEO* group of patients receiving neostigmine for reversal of cisatracurium-induced NMB, *SUG* group of patients receiving sugammadex for reversal of rocuronium-induced NMB, *M* male, *F* female, *BMI* body mass index, *ASA* American Society of Anesthesiologists physical status; neurovascular disease: history of transient ischemic attack, stroke, cerebral hemorrhage; respiratory disease: history of asthma, chronic obstructive pulmonary disease, or other diseases (restrictive lung diseases); cardiovascular disease: history of coronary artery disease, arrhythmia, heart failure, hypertension, disorders of the peripheral vascular system; abdominal disease: gastrointestinal disease (history of peptic ulcer, gastroesophageal reflux disease, irritable bowel syndrome, diverticular diseases, colitis, anal disease) and liver disease; other disease: rheumatic disease and musculoskeletal disease. *Previous KTx* previous kidney transplantation. Data are expressed as median [interquartile range, IQR] or number, *n* (%)

**Table 2:** Perioperative period

Medications	NEO (***n*** = 175)	SUG (***n*** = 175)	***P*** value
**Intraoperative period**			
Intravenous anesthesia, *n* (%)	62 (35.4)	56 (32)	0.572
Inhalational anesthesia, *n* (%)			
• Sevoflurane, *n* (%)	65 (37.1)	58 (33.1)	0.502
• Desflurane, *n* (%)	48 (27.4)	61 (34.9)	0.166
Fentanyl, *n* (%)	67 (38.3)	72 (41.1)	0.662
Remifentanil, *n* (%)	86 (49.1)	92 (52.6)	0.593
Ketamine, *n* (%)	20 (11.4)	15 (8.6)	0.476
Paracetamol, *n* (%)	153 (87.4)	159 (90.9)	0.391
Morphine, *n* (%)	27 (15.4)	25 (14.3)	0.881
Tramadol, *n* (%)	99 (56.6.4)	92(52.6.3)	0.520
Vasoactive drugs, *n* (%)	38 (21.7)	35 (20)	0.793
Droperidol, *n* (%)	39 (22.3.9)	42 (24)	0.800
Ondansetron, *n* (%)	149 (85.1)	141 (80.6)	0.321
Clonidine, *n* (%)	15 (8.6)	9 (5.1)	0.290
Pethidine, *n* (%)	10 (5.7)	7 (4.0)	0.620
Cisatracurium, mg [IQR]	36.9 [21.8-70.1]	0.0 [0.0-0.0]	< 0.001
Rocuronium, mg [IQR]	0.0 [0.0-0.0]	110.0 [60.0-220.0]	< 0.001
Atropine (%)	141 (80.6)	0.0 (0.0)	< 0.001
Neostigmine (%)	175.0 (100)	0.0 (0.0)	< 0.001
Neostigmine, μg/kg	29.9 [12.2-67.4]	0.0 [0.0-0.0]	< 0.001
Sugammadex 2 mg/kg (%)	0 (0.0)	101 (57.7)	< 0.001
Sugammadex 4 mg/kg (%)	0 (0.0)	74 (42.3)	< 0.001
Fluid total, ml	2100 [1800-2500]	2100 [1700-2400]	0.168
Surgery, min [IQR]	185 [155-237.5]	180 [150-225]	0.116
Anesthesia, min [IQR]	235 [210-292.5]	240 [205-285]	0.589
**Postoperative period**			
SpO_2_ at T15, %	99 [98-99]	99 [98-100]	0.117
HR at T15, beats/min [IQR]	81.0 [71-91]	82 [72.5-92.5]	0.271
SBP at T15, mmHg [IQR]	140.0 [130-154.5]	145 [128.5-157.5]	0.503
DBP at T15, mmHg [IQR]	80.0 [71.0-87.5.5]	79 [70.0-88.0]	0.348
pH at T15 [IQR]	7.36 [7.33-7.4]	7.36 [7.33-7.39]	0.671
PaO_2_ at T15, mmHg [IQR]	88.6 [76-103.2]	89.2 [79.5-101.4]	0.253
PaCO_2_ at T15, mmHg [IQR]	39.6 [36.2-42.4]	39.7 [36.4-43.6]	0.207
Pain NRS>3 at T15, *n* (%)	18 (10.3)	14 (8.0)	0.579
PONV NRS>3 at T15, *n* (%)	13 (7.4)	8 (4.6)	0.368
Hypoxemia, *n* (%)	11 (6.3)	1 (0.6)	0.006
Antihypertensive drugs, *n* (%)	16 (9.1)	9 (5.1)	0.212
PACU stay, min [IQR]	90 [60-105]	70 [60.0-90.0]	< 0.001
ICU admission, *n* (%)	14 [8.0]	1 [0.6]	0.001

*NMB* neuromuscular block, *NEO* group of patients receiving neostigmine for reversal of cisatracurium-induced NMB, *SUG* group of patients receiving sugammadex for reversal of rocuronium-induced NMB; vasoactive drugs: repeated administration of etilefrine or ephedrine, or dopamine infusion; surgery: time from skin incision to the placement of the last suture; anesthesia: time from tracheal intubation to tracheal extubation; *PACU* (post-anesthesia care unit): time from PACU admission to discharge to the surgical ward, *ICU* intensive care unit, *SpO*_*2*_ peripheral arterial blood oxygen saturation, *PaO*_*2*_ arterial partial pressure of oxygen, *PaCO*_*2*_ arterial partial pressure of carbon dioxide, *HR* heart rate, *SBP* systolic arterial blood pressure, *DBP* diastolic arterial blood pressure, *T15* 15 min after tracheal extubation, *NRS* numeric rating scale, *PONV* postoperative nausea and vomiting, *hypoxemia* oxygen desaturation (SaO_2_<90%) requiring treatment; antihypertensive drugs: clonidine, urapidil; amlodipine, labetalol; diltiazem. Data are expressed as median [interquartile range, IQR] or number, *n* (%)

**Fig. 1:**
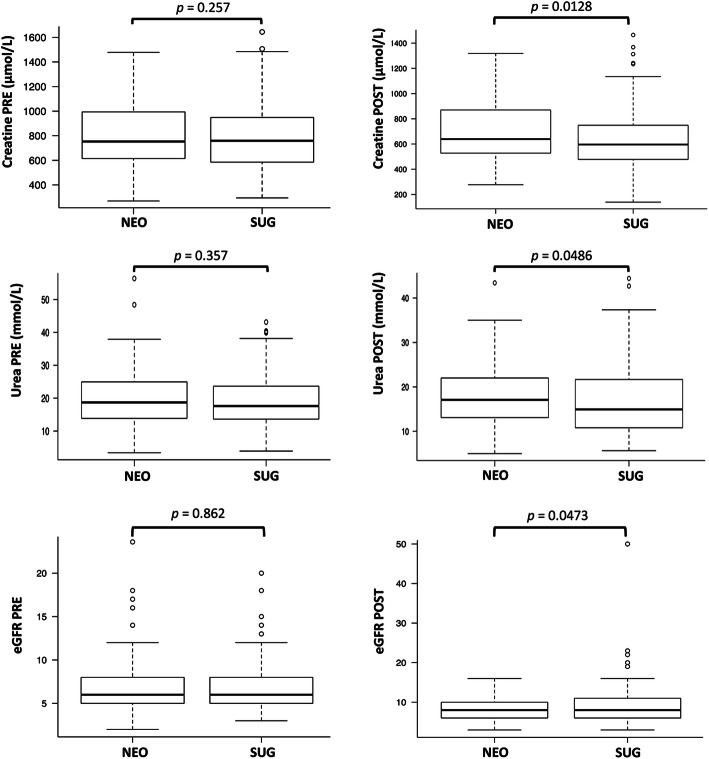
Box plots comparing sugammadex to neostigmine on kidney function before and after surgery. Boxes represent the median and IQR. “Whiskers” (minimum and maximum marks) represent values 1.5 times the IQR [(1st quartile–1.5 × IQR) and (3rd quartile+1.5 × IQR)]. *IQR* interquartile range, *p p* value with significance set at < 0.05, *NEO* group of patients receiving neostigmine for reversal of cisatracurium-induced neuromuscular block (NMB), *SUG* group of patients receiving sugammadex for reversal of rocuronium-induced NMB, *PRE* baseline value (before kidney transplantation), *POST* first measurement obtained the day of kidney transplantation, *creatinine and urea* serum creatinine and serum urea, *eGRF* estimated glomerular filtration rate

Regarding perioperative kidney function, serum creatinine (596.0 [478.0-749.0] vs 639.0 [527.7-870.0] μmol/L, *p* = 0.0128) and serum urea (14.9 [10.8-21.6] vs 17.1 [13.1-22.0] mmol/L, *p* = 0.0486) were lower, while eGFR (8.0 [6.0-11.0] vs 8.0 [6.0-10.0], *p* = 0.0473) was higher in the sugammadex group than in the neostigmine group after surgery (Table 3, Fig. 1). Serum urea remained significantly lower in the first 3 postoperative days (Table 3). No differences in kidney function were observed between the moderate and deep groups of sugammadex patients (Table 3). The proportion of patients with an increase in serum creatinine to > 44 μmol/L was higher in the sugammadex group than in the neostigmine group (first postoperative day: 40 [22.9%] vs 33 [18.9%], *p* = 0.430; fifth postoperative day: (15 [8.6%] vs 14 [8.0%], *p* = 1.0). However, the need for postoperative dialysis was higher in the neostigmine group than in the sugammadex group (21 [12%] vs 18 [10.3%], *p* = 0.734).

**Table 3:** Perioperative kidney function

Variable	Baseline	D0	D1	D2	D3	D4	D5
**Creatinine (μmol/L) [IQR]**							
NEO	753.0 [614.5-993.5]	639.0 [527.7-870.0]	558.0 [365.5-720.5]	397.0 [202.5-643.5]	253.0 [137.5-555.5]	190.0 [121.5-439.5]	170.0 [115.0-430.5]
SUG	759.0 [585.5-948.5]	596.0 [478.0-749.0] *	504.0 [370.0-658.0]	384.0 [170.0-596.5]	214.0 [126.5-496.5]	183.0 [117.0-433.5]	162.0 [107.5-379.0]
**Creatinine (μmol/L) [IQR]**							
SUG < 4 mg/kg (101 pts)	757.0 [592.0-944.0]	605.0 [502.0-744.0]	484.0 [367.0-642.0]	360.0 [170.0-611.0]	228.0 [134.0-500.0]	185.0 [126.0-437.0]	168.0 [121.0-399.0]
SUG ≥ 4 mg/kg (74 pts)	763.5 [573.0-953.0]	564.5 [461.5-798.5]	511.5 [378.5-680.5]	400.5 [173.5-569.0]	208.0 [119.7-494.0]	169.0 [104.7-422.0]	155.0 [100.0-362.0]
**Urea (mmol/L) [IQR]**							
NEO	18.7 [13.85-24.95]	17.1 [13.1-22.0]	17.5 [13.5-21.8]	17.9 [12.4-24.9]	16.4 [10.4-25.4]	15.8 [9.9-27.6]	14.7 [9.6-25.3]
SUG	17.6 [13.62-23.6]	14.9 [10.8-21.6] *	15.3 [11.3-19.8] *	15.9 [8.8-20.5] *	14.9 [7.5-20.7] *	14.2 [7.7-23.0]	12.9 [7.7-24.7]
**Urea (mmol/L) [IQR]**							
SUG < 4 mg/kg (101 pts)	17.44 [13.93-24.09]	15.48 [11.96-22.82]	15.9 [11.5-19.7]	16.4 [10.0-20.6]	14.7 [8-20.8]	14.4 [8.45-22.8]	14.37 [8.2-23.9]
SUG ≥ 4 mg/kg (74 pts)	17.6 [12.5-23.42]	14.3 [10.3-20.64]	14.45 [11.0-19.8]	14.7 [7.45-20.47]	15.1 [6.69-20.7]	13.35 [7.12-23.32]	12.5 [7.2-25.5]
**eGFR [IQR]**							
NEO	6.0 [5.0-8.0]	8.0 [6.0-10.0]	9.0 [7.0-14.0]	13.0 [7.5-29.0]	21.0 [9.0-43.5]	31.0 [11.0-51.0]	35.0 [12.2-53.7]
SUG	6.0 [5.0-8.0]	8.0 [6.0-11.0] *	10.0 [7.0-14.5]	14.0 [8.0-36.0]	23.0 [10.0-52.5]	32.0 [12.0-59.5]	36.0 [14.0-61.0]
**eGFR [IQR]**							
SUG < 4 mg/kg (101 pts)	6.0 [5.0-8.0]	8.0 [6.0-11.0]	10.0 [7.0-15.0]	15.0 [8.0-38.0]	23.0 [10.0-52.0]	32.0 [13.0-55.0]	36.0 [14.0-56.0]
SUG ≥ 4 mg/kg (74 pts)	6.0 [5.0-8.0]	8.0 [6.0-10.0]	9.0 [6.0-12.75]	14.0 [8.0-33.75]	26.0 [10.0-54.0]	33.0 [12.0-63.75]	37.0 [15.0-64.0]

*NMB* neuromuscular block, *NEO* group of patients receiving neostigmine for reversal of cisatracurium-induced NMB, *SUG* group of patients receiving sugammadex for reversal of rocuronium-induced NMB, *SUG <4 mg/kg and SUG ≥4 mg/kg* sugammadex dose per kg of body weight for reversal of moderate or deep NMB, respectively, *baseline* value before kidney transplantation, *DO-D5* values obtained the day of kidney transplantation (D0) and in each of the following five postoperative days (D1-D5), *eGRF* estimated glomerular filtration rate, *creatinine and urea* serum creatinine and serum urea, *eGRF* estimated glomerular filtration rate. Data are expressed as median [interquartile range, IQR]. (*) = significant *p* value (< 0.05) at intergroups analysis

**Fig. 2:**
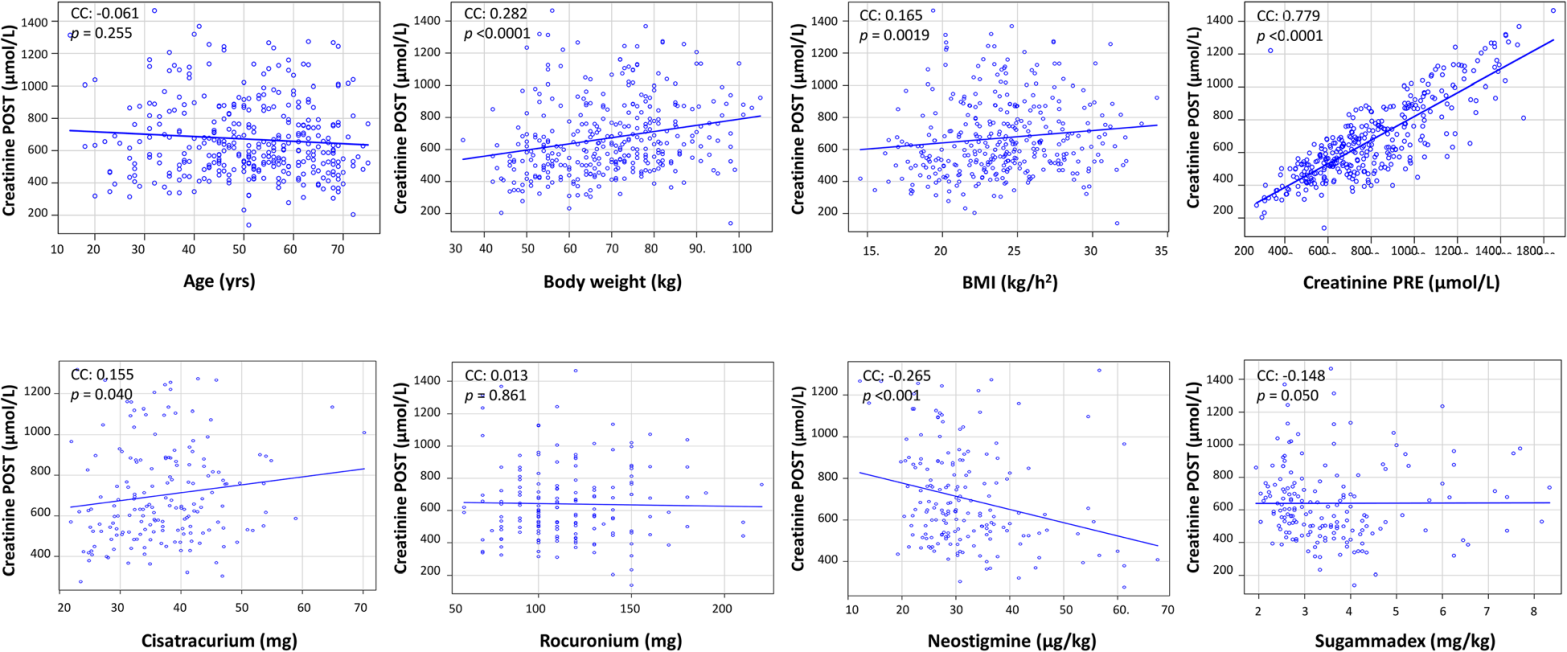
Correlation between patients’ characteristics, preoperative serum creatinine, and anesthetic drugs with postoperative serum creatinine. Drugs involved in neuromuscular block management were considered. *POST* first measurement of creatinine obtained after surgery the day of kidney transplantation, *mg* total dose, *mg/kg* dose in microgram (μg) or milligram (mg) per kilogram (kg) body weight, *BMI* body mass index. Spearman’s rank correlation tests each analysis. *CC* correlation coefficient, *p p* value with significance set at < 0.05

Body weight (CC = 0.282; *p* < 0.0001), height (CC = 0.281; *p* < 0.0001), body mass index (BMI) (CC = 0.165; *p* = 0.0019), preoperative serum creatinine (CC = 0.779; *p* < 0.0001), and neostigmine (CC = −0.265; *p* < 0.001) were correlated with postoperative serum creatinine (Fig. 2). No significant correlations were observed with other drugs involved in NMB management (Fig. 2).

During fitted multiple linear regression analysis, body weight (EC = 3.092; SE = 0.988; *t* = 3.127; *p* = 0.0019) and preoperative serum creatinine (EC = 0.706; SE = 0.027; t = −25.64; *p* < 0.0001) were the only variables associated with a higher level of postoperative serum creatinine, while rocuronium was associated with a lower level of postoperative serum creatinine (EC = −0.607; SE = 0.227; *t* = −2.666; *p* = 0.008).

## Discussion

In this study, sugammadex administered to reverse a rocuronium-induced NMB has been shown to improve recovery after kidney transplantation. Compared to the cisatracurium-neostigmine strategy, the rocuronium-sugammadex strategy resulted in lower incidence of postoperative respiratory events, faster discharge to the surgical ward, lower ICU admission, and better values of kidney function after surgery.

In patients with renal impairment, sugammadex was shown to effectively reverse both moderate (Staals et al. 2008; Staals et al. 2010) and deep (Cammu et al. 2012; de Souza et al. 2015; Panhuizen et al. 2015) rocuronium-induced NMB. No complications definitely, probably, or possibly related to the reversal drug have been reported (Staals et al. 2008; Staals et al. 2010; Cammu et al. 2012; de Souza et al. 2015; Panhuizen et al. 2015). In patients undergoing kidney transplantation, successful use of sugammadex for reversal of moderate rocuronium-induced NMB has been reported by retrospective observational studies (Unterbuchner, 2016; Ono et al. 2018; Arslantas and Cevik, 2019; Adams et al. 2020; Paredes et al., 2020; Vargas et al. 2020). Potential effects of sugammadex, or sugammadex-rocuronium complex, on renal function and the risk of postoperative recurrence of NMB are the main concerns about the rocuronium-sugammadex strategy in subjects with ESRD, including those undergoing kidney transplantation.

After administration, sugammadex (and sugammadex-rocuronium complexes) is renally excreted (Bom et al. 2009; Staals et al. 2010). In a pharmacokinetic study, excretion of (14)C-labeled sugammadex was rapid, with around 70% of the dose excreted within 6 h and around 90% within 24 h. Consequently, the major route of elimination of rocuronium changes from the hepatic to the renal route (Peeters et al. 2011). In patients with ESRD, total plasma clearance of sugammadex was 17 times lower and mean elimination half-life was 16 times higher in the renal failure group compared to control (Staals et al. 2010). Therefore, administration of sugammadex after rocuronium results in lengthened exposure of renal glomeruli and tubules to sugammadex and sugammadex-rocuronium complexes, leading to their hypothesized role in the renal impairment after surgery (Bostan et al. 2011). However, cyclodextrins are highly water-soluble cyclic oligosaccharides without intrinsic biological activity; it is therefore unlikely that side effects will occur after administration (Staals et al. 2011). Toxicity studies on γ-cyclodextrins have shown that the drugs are well tolerated and elicit no toxicological effects (Munro et al. 2004). Also, sugammadex, belonging to the family of γ-cyclodextrins, is biologically inactive and, administered at the recommended dose, has been shown to be well tolerated in patients with renal impairment (Staals et al. 2008; Staals et al. 2010; Cammu et al. 2012; de Souza et al. 2015; Adams et al. 2020; Paredes et al., 2020). In an experimental study, only sugammadex administered at a higher dose (96 mg/kg) than recommended (≤ 16 mg/kg) resulted in a significant increase of histopathological changes in the rat kidney (dilatation, vascular vacuolation and hypertrophy, lymphocyte infiltration, and tubular cell sloughing) compared to the control group (Bostan et al. 2011). Similar findings were reported in streptozotocin-induced diabetic rats. Diabetic nephropathy predisposes to changes in kidney tissues, including inflammation, degeneration, necrosis, tubular dilatation, tubular cell degeneration, dilatation in Bowman’s space, tubular hyaline casts, and lymphocyte infiltration. In renal tissue samples, a significant increase in histopathological changes was found after sugammadex 96 mg/kg but not sugammadex 16 mg/kg treatment compared to diabetic control (Kip et al. 2015). These results suggest that, at recommended doses, sugammadex does not significantly impact renal function (Bostan et al. 2011), also in case of diabetic nephropathy (Kip et al. 2015). In a dose-finding and safety study in adult patients, abnormal levels of N-acetyl-glucosaminidase were only found in 5 of 20 patients included in the intent-to-treat population and safety population (Sorgenfrei et al. 2006). However, changes in urinalysis were reported in the active treatment groups (sugammadex 0.5-4.0 mg/kg) as well as in the placebo group but were not considered to be clinically relevant (Sorgenfrei et al. 2006).

The impact on renal function of sugammadex compared to neostigmine for reversal of NMB also deserves consideration. A study designed to evaluate the cytotoxic, genotoxic, and apoptotic effects of different dosages of both reversal drugs on human embryonic renal (HEK-293) cells showed that neostigmine administered in vitro at 50, 100, 250, and 500 μg/mL had greater cytotoxic, genotoxic, and apoptotic effects on HEK-293 cells than the equivalent dosages of sugammadex (Büyükfırat et al. 2018). In adult patients undergoing desflurane/opioid anesthesia who received neostigmine 40 μg/kg and sugammadex 4 mg/kg to reverse rocuronium-induced NMB, renal glomerular filtration and tubular functions were minimally affected. However, these effects were greater with neostigmine than with sugammadex. No significant changes were observed for serum creatinine and urea levels between the two groups. Instead, in urinalysis, the postoperative value of cystatin C, a specific marker of glomerular function, was found to be significantly higher in the neostigmine group compared to the sugammadex group (Isik et al. 2016). Comparing the rocuronium-sugammadex strategy to the cisatracurium-neostigmine strategy in adult patients, significant differences were found only in urinalysis, with N-acetyl-glucosaminidase higher in the rocuronium-sugammadex group, and β_2_-microglobulin higher in the cisatracurium-neostigmine group (Flockton et al. 2008).

A comparison of the rocuronium-sugammadex and cisatracurium-neostigmine strategies was retrospectively evaluated in kidney transplantation, but the sample size was not large enough to draw a conclusion on the impacts of sugammadex and neostigmine on renal function in such population of patients, and no data are included on sugammadex administered to reverse deep rocuronium-induced NMB (Vargas et al. 2020). This study confirmed the advantage of the rocuronium-sugammadex over the cisatracurium-neostigmine strategy not only in improving postoperative kidney function (Vargas et al. 2020) but also in promoting a better general recovery, independently from the level of NMB at the end of surgery. This may be explained by different impacts of the two reversal drugs on renal function (Munro et al. 2004; Sorgenfrei et al. 2006; Flockton et al. 2008; Staals et al. 2011; Bostan et al. 2011; Kip et al. 2015; Isik et al. 2016; Büyükfırat et al. 2018; Vargas et al. 2020), a restoration of glomerular filtration after surgery that minimizes the stasis of the sugammadex (and rocuronium-sugammadex complex) in the glomeruli and tubules (Bostan et al. 2011; Kip et al. 2015; Vargas et al. 2020), and a potential protective effect of sugammadex against ischemia-reperfusion injury (Vargas et al. 2020). In an experimental study, sugammadex 16 mg/kg and 100 mg/kg, administered to evaluate the benefit of cyclodextrins against transient global cerebral ischemia, showed a dose-dependent neuroprotective effect in a transient global cerebral ischemia/reperfusion rat model (Ozbilgin et al. 2016). In the postoperative period, the transient increase of serum urea, which peaked on the third day after surgery, may be due to the catabolic effects of corticosteroids administered perioperatively to prevent graft rejection and of diuretics (Vargas et al. 2020).

Serum creatinine level significantly decreased over time after kidney transplantation. The recipient’s age was negatively associated with their postoperative serum creatinine values. No significant association was found between serum creatinine levels and the recipient’s BMI, gender, or history of dialysis (Younespour et al. 2016). On the basis of our analysis, postoperative serum creatinine significantly depends on preoperative values. Most importantly, the drugs involved in NMB management had no effect on serum creatinine. A positive association has been shown between serum creatinine levels and graft failure, which means that graft failure is more likely to occur in patients with higher postoperative serum creatinine levels (Younespour et al. 2016; Maraghi et al. 2016). A one-unit increase in the serum creatinine level was found to be associated with a four- (Younespour et al. 2016) or five-times (Maraghi et al. 2016) higher risk of graft failure.

A high affinity of rocuronium to sugammadex allows the guest-host complex to exist in equilibrium with a very high association rate (an association constant of 10^7^ M^−1^) and a very low dissociation rate, so the complex is tight, and recurrence of NMB is highly unlikely (Bom et al. 2009). The absence of recurrences of NMB observed in our large cohort of patients supports the safety of the rocuronium-sugammadex strategy in kidney transplantation and confirms the findings from other observational studies. Ono et al. (2018) reported a successful use of sugammadex in 99 consecutive patients undergoing kidney transplantation, Adams et al. (2020) in 48 patients, and Vargas et al. (2020) in 30 patients, without recurrences of NMB. Interestingly, among 158 patients with ESRD undergoing a surgical procedure, sugammadex was administered to 24 patients (18%) who had initially been reversed with a standard dose of neostigmine (70 μg/kg up to a maximum dose of 5 mg) for residual NMB, with immediate and full reversal of muscle strength loss and successful tracheal extubation at the end of surgery (Adams et al. 2020). A more favorable recovery after sugammadex compared to neostigmine is supported by the literature. Recovery to TOFR > 1.0 is recommended when acceleromyography is used (Eikermann et al. 2007). Although TOFR ≥ 0.9 indicates adequate recovery from NMB, it does not necessarily mean that neuromuscular function has returned to normal and may increase the risk of upper airway obstruction, hypoventilation, hypoxia, and other postoperative respiratory complications (Eikermann et al. 2007; Blobner et al. 2020). Tracheal extubation in patients with TOFR > 0.95 has been shown to reduce the adjusted risk of postoperative pulmonary complications compared to extubation at TOFR > 0.9 (Blobner et al. 2020). Both quantitative monitoring of neuromuscular function and an appropriate dosage of reversal drug titrated to the level of NMB concur with a full reversal (TOFR ≥ 1.0) and an improvement of patient outcomes (Eikermann et al. 2007; Blobner et al. 2020). Compared to neostigmine, sugammadex has been associated with lower risk of postoperative complications (Carron M, Baratto F 2016) and a better recovery profile that allows faster discharge from the operating theater and PACU (Carron et al. 2020) and reduced risk of ICU admission (Carron M, Baratto F 2016).

This study has some limitations. First, it is not a randomized controlled study and therefore has the drawbacks of all observational studies. The evidence of non-inferiority may warrant a specific prospective, randomized clinical trial. Second, the temporal factor may be a potential bias, even if the majority of patients were recruited close to the change in strategy for NMB management and no changes in the surgical team or perioperative care were adopted in the study period. Third, we were unable to compare the exact values of TOFR ≥ 0.90 before extubation, which might cause an unmatched level of recovery after reversal of neuromuscular block at the time of extubation between the two study groups. Fourth, more specific markers (e.g., cystatin C, N-acetyl-glucosaminidase, α_1_-microglobulin, β_2_-microglobulin) were not available for a targeted analysis of postoperative renal function.

In conclusion, sugammadex should be considered for reversal of rocuronium-induced NMB in patients undergoing kidney transplantation.

## Data Availability

The datasets used and analyzed during the current study are available from the corresponding author on reasonable request.

## Abbreviations

aOR: Adjusted odds ratio
BMI: Body mass index
CC: Correlation coefficient
CrCl: Creatinine clearance
DBP: Diastolic arterial blood pressures
EC: Estimate coefficient
eGFR: Estimated glomerular filtration rate
ESRD: End-stage renal disease
HR: Heart rate
ICU: Intensive care unit
NMB: Neuromuscular block
NMBA: Neuromuscular block agent
NRS: Numeric rating scale
PACU: Post-anesthesia care unit
PaCO_2_: Arterial partial pressures of carbon dioxide
PaO_2_: Arterial partial pressures of oxygen
PONV: Postoperative nausea and vomiting
SaO_2_: Peripheral arterial blood oxygen saturation
SBP: Systolic arterial blood pressure
SE: Standard error
STROBE: STrengthening the Reporting of Observational studies in Epidemiology
TOFR: Train-of-four ratio

## References

[CR1] Adams DR, Tollinche LE, Yeoh CB, Artman J, Mehta M, Phillips D, Fischer GW, Quinlan JJ, Sakai T (2020). Short-term safety and effectiveness of sugammadex for surgical patients with end-stage renal disease: a two-centre retrospective study. Anaesthesia.

[CR2] Arslantas R, Cevik BE (2019). Retrospective investigation of grafted kidney function after reversal of neuromuscular blockade using neostigmine or sugammadex. Transplant Proc.

[CR3] Blobner M, Hunter JM, Meistelman C, Hoeft A, Hollmann MW, Kirmeier E, Lewald H, Ulm K (2020). Use of a train-of-four ratio of 0.95 versus 0.9 for tracheal extubation: an exploratory analysis of POPULAR data. Br J Anaesth.

[CR4] Bom A, Hope F, Rutherford S, Thomson K (2009). Preclinical pharmacology of sugammadex. J Crit Care.

[CR5] Bostan H, Kalkan Y, Tomak Y, Tumkaya L, Altuner D, Yılmaz A, Erdivanli B, Bedir R (2011). Reversal of rocuronium-induced neuromuscular block with sugammadex and resulting histopathological effects in rat kidneys. Ren Fail.

[CR6] Brull SJ, Kopman AF (2017). Current status of neuromuscular reversal and monitoring: challenges and opportunities. Anesthesiology.

[CR7] Büyükfırat E, Koyuncu İ, Karahan MA, Binici O, Altay N, Kirmit A, Gönel A (2018). Comparison of the cytotoxic, genotoxic and apoptotic effects of sugammadex and neostigmine on human embryonic renal cell (HEK-293). Cell Mol Biol (Noisy-le-grand).

[CR8] Cammu G, Van Vlem B, van den Heuvel M, Stet L, el Galta R, Eloot S (2012). Dialysability of sugammadex and its complex with rocuronium in intensive care patients with severe renal impairment. Br J Anaesth.

[CR9] Carron M, Baratto F, Zarantonello F, Ori C (2016). Sugammadex for reversal of neuromuscular blockade: a retrospective analysis of clinical outcomes and cost-effectiveness in a single center. Clinicoecon Outcomes Res.

[CR10] Carron M, Linassi F, De Cassai A (2020). Role of sugammadex in accelerating postoperative discharge: an updated meta-analysis. J Clin Anesth.

[CR11] Carron M, Zarantonello F, Tellaroli P, Ori C (2016). Efficacy and safety of sugammadex compared to neostigmine for reversal of neuromuscular blockade: a meta-analysis of randomized controlled trials. J Clin Anesth.

[CR12] De Gasperi A, Feltracco P, Ceravola E, Mazza E (2014). Pulmonary complications in patients receiving a solid-organ transplant. Curr Opin Crit Care.

[CR13] de Souza CM, Tardelli MA, Tedesco H, Garcia NN, Caparros MP, Alvarez-Gomez JA, de Oliveira Junior IS (2015). Efficacy and safety of sugammadex in the reversal of deep neuromuscular blockade induced by rocuronium in patients with end-stage renal disease: a comparative prospective clinical trial. Eur J Anaesthesiol.

[CR14] Della Rocca G, Pompei L, Coccia C, Costa MG, Cecchini V, Vilardi V (2003). Atracurium, cisatracurium, vecuronium and rocuronium in patients with renal failure. Minerva Anestesiol.

[CR15] Eikermann M, Vogt FM, Herbstreit F, Vahid-Dastgerdi M, Zenge MO, Ochterbeck C, de Greiff A, Peters J (2007). The predisposition to inspiratory upper airway collapse during partial neuromuscular blockade. Am J Respir Crit Care Med.

[CR16] EMA. Bridion (sugammadex sodium) 100 mg/mL: summary of product characteristics. In: . Available from URL: http://www.ema.europa.eu/docs/en_GB/document_library/EPAR_-_Product_Information/human/000885/WC500052310.pdf (Accessed January 25, 2021).

[CR17] Flockton EA, Mastronardi P, Hunter JM, Gomar C, Mirakhur RK, Aguilera L, Giunta FG, Meistelman C, Prins ME (2008). Reversal of rocuronium-induced neuromuscular block with sugammadex is faster than reversal of cisatracurium-induced block with neostigmine. Br J Anaesth.

[CR18] Gameiro J, Agapito Fonseca J, Jorge S, Lopes JA (2018). Acute kidney injury definition and diagnosis: a narrative review. J Clin Med.

[CR19] Isik Y, Palabiyik O, Cegin BM, Goktas U, Kati I (2016). Effects of sugammadex and neostigmine on renal biomarkers. Med Sci Monit.

[CR20] Kellar CA (2015). Solid organ transplantation overview and delection criteria. Am J Manag Care.

[CR21] Kheterpal S, Vaughn MT, Dubovoy TZ, Shah NJ, Bash LD, Colquhoun DA, Shanks AM, Mathis MR, Soto RG, Bardia A, Bartels K, McCormick PJ, Schonberger RB, Saager L (2020). Sugammadex versus neostigmine for reversal of neuromuscular blockade and postoperative pulmonary complications (STRONGER): a multicenter matched cohort analysis. Anesthesiology.

[CR22] Kip G, Turgut HC, Alkan M, Aydin ME, Erbatur ME, Kiraz HA, Kartal S, Boyunaga H, Comu FM, Erdem O, Arslan M, Unal Y (2015). The effects of low and high doses of sugammadex on kidney tissue in streptozotocin-induced diabetic rats. Bratisl Lek Listy.

[CR23] Kirmeier E, Eriksson LI, Lewald H, Jonsson Fagerlund M, Hoeft A, Hollmann M, Meistelman C, Hunter JM, Ulm K, Blobner M, Abad Gurumeta A, Abernethy C, Abigail P, Achaibar K, Adam E, Afshari A, Agudelo Montoya ME, Akgün FN, Aletti G, Alkış N, Allan K, Allan A, Allaouchiche B, Allcock C, Almasy E, Amey I, Amigoni M, Andersen E, Andersson P, Anipchenko N, Antunes P, Armstrong E, Aslam TN, Aslin B, Assunção JP, Ausserer J, Avvai M, Awad N, Ayas Montero B, Ayuso M, Azevedo P, Badarau V, Badescu R, Baiardo Redaelli M, Baird C, Baird Y, Baker T, Balaji P, Bălan C, Balandin A, Balescu-Arion C, Baliuliene V, Baltasar Isabel J, Baluch SN, Bandrabur D, Bankewitz C, Barber K, Barbera F, Barcraft-Barnes H, Barletti V, Barnett G, Baron K, Barros A, Barsan V, Bartlett P, Batistaki C, Baumgarten G, Baytas V, Beauchamp N, Becerra Cayetano IA, Bell S, Bellandi M, Belletti A, Belmonte Cuenca J, Benitez-Cano A, Beretta L, Berger M, Bergmann N, Bergmark K, Bermudez Lopez M, Bernotaite M, Beurskens C, Bidd H, Bifulco F, Bignami E, Bilic A, Bilskiene D, Bischoff P, Bishop L, Bjonness T, Blaylock H, Blethyn K, Blincoe T, Blokhin I, Blunt N, Boer C, Bois G, Bonicolini E, Booth J, Borecka-Kedzierska M, Borstnar K, Borys M, Boselli E, Bouvet L, Bouwman A, Bowen L, Bowrey S, Boxall L, Božić T, Bradley T, Branco T, Brazzi L, Brazzoni M, Brear T, Brogly N, Brohi F, Broms J, Bubliauskas A, Bucolo GE, Buerkle H, Buggy D, Buhre W, Bukauskas T, Butturini F, Byttner A, Cabrera Díaz I, Calderon A, Calhau R, Callejo A, Cammu G, Campesato M, Can ÖS, Candeias M, Cantor A, Carise E, Carmona C, Carreteiro J, Carrieri C, Carter A, Casal M, Casanova I, Cascella M, Casero LM, Casiraghi GM, Castelo-Branco L, Castro Arranz C, Cernea DD, Cervantes J, Chandler B, Charnock R, Chatzimicali A, Chinery E, Chishti A, Chondhury P, Christie E, Christodoudiles G, Ciardo S, Cimpeanu L, Cindea I, Cinnella G, Clark S, Clayton M, Cocu S, Collyer T, Colvin C, Cope S, Copeta F, Copotoiu SM, Correia de Barros F, Corso RM, Cortegiani A, Costa G, Cowton A, Cox N, Craig J, Cricca V, Cronin J, Cunha M, Cuomo A, Curley K, Czuczwar M, Dabrowska D, Damster S, Danguy des Déserts M, Daniliuc A, Danninger T, Darwish I, Dascalu C, Davies K, Davies S, de Boer H, de Flaviis A, de Selincourt G, Deana C, Debaene B, Debreceni G, Dedhia J, Delgado Garcia I, Della Rocca G, Delroy-Buelles L, Desai T, Dhillon P, di Giacinto I, di Mauro P, Diaz Gomez TV, Dimitrovski A, Dinic V, Dîrzu DS, Divander MB, Dolinar J, Domingues S, Doolan J, Downes C, Dragoescu NA, Droc G, Dum E, Dumitrescu A, Duncan L, Dzurňáková P, Eberl S, Edwards J, Edwards M, Ekelund K, Ekengren P, Elghouty E, Ellerkmann R, Ellis H, Elme A, Ernst T, Errando CL, Estenes S, Ewaldsson C, Farid N, Featherstone J, Febres D, Fedorov S, Feggeler J, Feijten P, Fellmann T, Fernandez Candil J, Fernandez Castineira A, Fernández Castineira J, Fernando A, Ferrando C, Ferreira L, Ferreira P, Feyling A, Filipescu D, Fleischer A, Floris L, Foerster U, Fox B, Franke U, Frasca D, Frey C, Frost V, Fullin G, Fumagalli J, Furneval J, Fusari M, Gallacher S, Galushka S, Gambale G, Gambino I, Garcia-Perez ML, Garg S, Garlak J, Gavranovic Z, Gavrilov R, Gaynor L, Gecaj Gashi A, Georghiou M, Gerjevic B, Gferer G, Giarratano A, Gibson A, Gievski V, Giles J, Gillberg L, Gilowska K, Gilsanz Rodriguez F, Gioia A, Giovannoni C, Girotra V, Gkinas D, Gkiokas G, Godoroja D, Goebel U, Goel V, Gonzalez M, Goranovic T, Gornik-Wlaszczuk E, Gosavi S, Gottfridsson, Gottschalk A, Granell M, Granstrom A, Grassetto A, Greenwood A, Grigoras I, Grintescu I, Gritsan A, Gritsan G, Grynyuk A, Guadagnin GM, Guarnieri M, Güçlü Ç, Guerrero Diez M, Gunenc F, Günther U, Gupta P, Guttenthaler V, Hack Y, Hafisayena A, Hagau N, Haldar J, Hales D, Hancı V, Hanna-Jumma S, Harazim H, Harlet P, Harper D, Harris B, Harvey O, Hashimi M, Hawkins L, Hayes C, Heaton J, Heier T, Helliwell L, Hemmes S, Henderson K, Hermanides J, Hermanns H, Herrera Hueso B, Hestenes S, Hettiarachchi R, Highgate J, Hodgson K, Hoelbling D, Holland J, Horhota L, Hormis A, Hribar R, Hua A, Humphreys S, Humphries R, Humpliková S, Hunt J, Husnain A, Hussein A, Hyams B, Iannuccelli F, Ilette K, Ilyas C, Inan T, India I, Ionițăv V, Irwin F, Jain V, Janez B, Jankovic R, Jenkins S, Jenko M, Jimenez R, Jiménez Gomez B, Joachim S, Joelsson-Alm E, John J, Jonikaite L, Jovic M, Jungwirth B, Junke E, Kabakov B, Kadaoui SD, Kanski A, Karadag S, Karbonskiene A, Karjagin J, Kasnik D, Katanolli F, Katsika E, Kaufmann K, Keane H, Kelly M, Kent M, Keraitiene G, Khudhur A, Khuenl-Brady K, Kidd L, King S, Kirchgäßner K, Klancir T, Klucniks A, Knotzer J, Knowlden P, Koers L, Kompan J, Koneti KK, Kooij F, Koolen E, Koopman - van Gemert AWMM, Kopp K, Korfiotis D, Korolkov O, Kosinová M, Köstenberger M, Kotzinger O, Kovačević M, Kranke P, Kranke E, Kraus C, Kraus S, Kubitzek C, Kucharski R, Kucukguclu S, Kudrashou A, Kumar V, Kummen L, Kunit C, Kushakovsky V, Kuvaki B, Kuzmanovska B, Kyttari A, Landoni G, Lau G, Lazarev K, Legett S, Legrottaglie AM, Leonardi S, Leong M, Lercher H, Leuvrey M, Leva B, Levstek M, Limb J, Lindholm E, Linton F, Liperi C, Lipski F, Lirk P, Lisi A, Lišková K, Lluch Oltra A, Loganathan V, Lombardi S, Lopez E, Lopez Rodríguez M, Lorenzini L, Lowicka M, Lugovoy A, Luippold M, Lumb A, Macas A, Macgregor M, Machado H, Maciariello M, Madeira I, Maitan S, Majewski J, Maldini B, Malewski G, Manfredini L, Männer O, Marchand B, Marcu A, Margalef J, Margarson M, Marinheiro L, Markic A, Markovic Bozic J, Marrazzo F, Martin J, Martin Ayuso M, Martinez E, Martino EA, Martinson V, Marusic-Gaser K, Mascarenhas C, Mathis C, Matsota P, Mavrommati E, Mazul Sunko B, McCourt K, McGill N, McKee R, Meço BC, Meier S, Melbourne S, Melbybråthen G, Meli A, Melia A, Melotti RM, Menga MR, Mercer P, Merotra S, Mescolini S, Metterlein T, Michalov M, Michlig S, Midgley S, Milić M, Milojevic M, Miñana A, Minto G, Mirabella L, Mirea L, Mittelstädt L, Moeglen A, Moise A, Mokini Z, Molin A, Moltó L, Monea MC, Montalto F, Montgomery J, Montgomery C, Montillo G, Moore S, Moore F, Moreira Z, Moreno T, Moreno R, Moret E, Moreton S, Morgan M, Moro Velasco C, Morri D, Moull A, Moura F, Mráz P, Mrozek K, Mukhtar K, Muniyappa S, Murray H, Murthy BVS, Mushambi M, Nadolski M, Nardelli P, Nardin G, Navarro Pérez R, Naveiro A, Negri M, Nesek Adam V, Neskovic V, Neuwersch S, Neves M, Nguyen B, Ní Eochagáin A, Nicholas C, Nightingale J, Norrie K, Novak-Jankovic V, Novakova A, Novillo M, Numan S, Oduro-Dominah L, Oldner A, Oliveira I, Ologoiu D, Oloktsidou I, O'Reilly R, Orlando A, Ovezov A, Ozbilgin S, Paal P, Padin Barreiro L, Palugniok R, Papaioannou A, Papapostolou K, Paranthaman P, Pardey Bracho G, Parente S, Parfeni A, Pasin L, Passey S, Pastor E, Patch S, Patil A, Paunescu MA, Pehboeck D, Pereira M, Pereira C, Perez Caballero P, Pérez García A, Pérez Soto A, Perez Tejero G, Perez-Cerda F, Pesenti A, Petta R, Philippe S, Pickering D, Pico Veloso J, Pina P, Pinho-Oliveira V, Pinol S, Pinto R, Pistidda L, Pitterle M, Piwowarczyk P, Plotnikova O, Pohl H, Poldermann J, Polkovicová L, Pompei L, Popescu M, Popović R, Pota V, Potocnik M, Potręć B, Potter A, Pramod N, Prchalova M, Preckel B, Pugh R, Pulletz M, Radoeshki A, Rafi A, Ragazzi R, Raineri Santi M, Rajamanickam T, Rajput Z, Ramachandran R, Ramasamy R, Ramessur S, Rao R, Rasmussen A, Rato A, Razaque U, Real Navacerrada MI, Reavley C, Reid J, Reschreiter H, Rial E, Ribas Carrasco P, Ribeiro S, Rich N, Richardson L, Rimaitis K, Rimaitis M, Ringvold EM, Ripke F, Ristescu I, Ritchie K, Ródenas F, Rodrigues P, Rogers E, Rogerson D, Romagnoli S, Romero E, Rondovic G, Rose BO, Roth W, Rotter MT, Rousseau G, Rudjord A, Rueffert H, Rundgren M, Rupprecht K, Rushton A, Russotto V, Rypulak E, Ryszka M, Sà J, Sà Couto P, Saby S, Sagic J, Saleh O, Sales G, Sánchez Sánchez Y, Sanghera S, Şanli Karip C, Santiveri Papiol FJ, Santos S, Sarno S, Saul D, Saunders D, Savic N, Scalco L, Scanlon D, Schaller S, Schax C, Scheffer GJ, Schening A, Schiavone V, Schmidt-Ehrenberg F, Schmidt-Mutter C, Schönberg C, Schopflin C, Schreiber JU, Schultz M, Schurig M, Scott C, Sebestian S, Sehgal S, Sem V, Semenas E, Serafini E, Serchan P, Shields M, Shobha R, Shosholcheva M, Siamansour T, Siddaiah N, Siddiqi K, Sinclair R, Singh P, Singh R, Sinha A, Sinha A, Skinner A, Smee E, Smekalova O, Smith N, Smith T, Smitz C, Smole D, Sojčić N, Soler Pedrola M, Somanath S, Sonksen J, Sorella MC, Sörmus A, Soro M, Soto C, Spada A, Spadaro S, Spaeth J, Sparr H, Spielmann A, Spindler-Vesel A, Stamelos M, Stancombe L L, Stanculescu A, Standl T, Standley T, Stanek O, Stanisavljević S, Starczewska M, Stäuble C, Steen J, Stefan OM, Stell E, Stera C, Stevens M, Stoerckel M, Stošić B, Stourac P, Stroumpoulis K, Struck R, Suarez de la Rica A, Sultanpori A, Sundara Rajan R, Suying O, Svensen C, Swan L, Syrogianni P, Sysiak J, Szederjesi J, Taddei S, Tan Hao E, Tanou V, Tarabová K, Tardaguila Sancho P, Tarroso M, Tartaglione M, Taylor E, Tbaily L, Telford R, Terenzoni M, Theodoraki K, Thornley H, Tiganiuc L, Toim H, Tomescu D, Tommasino C, Toni J, Toninelli A, Toretti I, Townley S, Trepenaitis D, Trethowan B, Tsaousi G, Tsiftsi A, Tudor A, Turan G, Turhan SÇ, Unic-Stojanovic D, Unterbuchner C, Unzueta C, Uranjek J, Ursic T, Vaida S, Valldeperas Ferrer S, Valldeperas Hernandez MI, Valsamidis D, van Beek R, van dasselaer N, van der Beek T, van Duivenvoorde Y, van Klei WA, van Poorter F, van Zaane B, van Zundert T, van Zyl R, Vargas Munoz AM, Varsani N, Vasconcelos P, Vassilakis G, Vecchiatini T, Vecera L, Vercauteren M, Verdouw B, Verheyen V, Verri M, Vicari Sottosanti LG, Vico M, Vidal Mitjans P, Vilardi A, Vissicchio D, Vitale G, Vitković B, Vizcaychipi MP, Voicu A, Voje M, Volfová I, Volta CA, von Lutterotti T, von Tiesenhausen A, Vrecic-Slabe S, Vukcevic D, Vukovic R, Vullo PA, Wade A, Wallberg H, Wallden J, Wallner J, Walther Sturesson L, Watson D, Weber S, Wegiel Leskiewiq A, Weller D, Wensing C, Werkmann M, Westberg H, Wikström E, Williams B, Williams B, Wilson R, Wirth S, Wittmann M, Wood L, Wright S, Zachoval C, Zambon M, Zampieri S, Zampone S, Zangrillo A, Zani G, Zavackiene A, Zieglerder R, Zonneveldt H, Zsisku L, Zucker TP, Żukowski M, Zuleika M, Zupanĕiĕ D (2019). POPULAR Contributors. Post-anaesthesia pulmonary complications after use of muscle relaxants (POPULAR): a multicentre, prospective observational study. Lancet Respir Med.

[CR24] Kork F, Balzer F, Spies CD, Wernecke KD, Ginde AA, Jankowski J, Eltzschig HK (2015). Minor postoperative increases of creatinine are associated with higher mortality and longer hospital length of stay in surgical patients. Anesthesiology.

[CR25] Maraghi E, Rahimi Foroushani A, Younespour S, Rostami Z, Einollahi B, Eshraghian MR, Akhoond MR, Mohammad K (2016). Longitudinal assessment of serum creatinine levels on graft survival after renal transplantation: joint modeling approach. Nephrourol Mon.

[CR26] Martinez BS, Gasanova I, Adesanya AO (2013). Anesthesia for kidney transplantation-a review. J Anesth Clin Res.

[CR27] Miskovic A, Lumb AB (2017). Postoperative pulmonary complications. Br J Anaesth.

[CR28] Mittel AM, Wagener G (2017). Anesthesia for kidney and pancreas transplantation. Anesthesiol Clin.

[CR29] Munro IC, Newberne PM, Young VR, Bär A (2004). Safety assessment of gamma-cyclodextrin. Regul Toxicol Pharmacol.

[CR30] Ono Y, Fujita Y, Kajiura T, Okawa H, Nakashima J, Isobe H, Fujiwara Y (2018). Efficacy and safety of sugammadex in patients undergoing renal transplantation. JA Clin Rep.

[CR31] Ozbilgin S, Yılmaz O, Ergur BU, Hancı V, Ozbal S, Yurtlu S, Gunenc SF, Kuvaki B, Kucuk BA, Sisman AR (2016). Effectiveness of sugammadex for cerebral ischemia/reperfusion injury. Kaohsiung J Med Sci.

[CR32] Panhuizen IF, Gold SJ, Buerkle C, Snoeck MM, Harper NJ, Kaspers MJ (2015). Efficacy, safety and pharmacokinetics of sugammadex 4 mg kg^-1^ for reversal of deep neuromuscular blockade in patients with severe renal impairment. Br J Anaesth.

[CR33] Paredes S, Porter SB, Porter IE, Renew JR (2020). Sugammadex use in patients with end-stage renal disease: a historical cohort study. Can J Anaesth.

[CR34] Peeters P, Passier P, Smeets J, Zwiers A, de Zwart M, van de Wetering-Krebbers S, van Iersel M, van Marle S, van den Dobbelsteen D (2011). Sugammadex is cleared rapidly and primarily unchanged via renal excretion. Biopharm Drug Dispos.

[CR35] Sorgenfrei IF, Norrild K, Larsen PB, Stensballe J, Ostergaard D, Prins ME (2006). Reversal of rocuronium-induced neuromuscular block by the selective relaxant binding agent sugammadex: a dose-finding and safety study. Anesthesiology.

[CR36] Staals LM, de Boer HD, van Egmond J, Hope F, van de Pol F, Bom AH, Driessen JJ, Booij LHDJ (2011). Reversal of rocuronium-induced neuromuscular block by sugammadex is independent of renal perfusion in anesthetized cats. J Anesth.

[CR37] Staals LM, Snoeck MM, Driessen JJ, Flockton EA, Heeringa M, Hunter JM (2008). Multicentre, parallel-group, comparative trial evaluating the efficacy and safety of sugammadex in patients with end-stage renal failure or normal renal function. Br J Anaesth.

[CR38] Staals LM, Snoeck MM, Driessen JJ, van Hamersvelt HW, Flockton EA, van den Heuvel MW (2010). Reduced clearance of rocuronium and sugammadex in patients with severe to end-stage renal failure: a pharmacokinetic study. Br J Anaesth.

[CR39] Unterbuchner C (2016). Use of rocuronium and sugammadex in renal transplantation: problems that must be considered. Eur J Anaesthesiol.

[CR40] Vargas M, Buonanno P, Sica A, Sabatella E, D’Alessio FP, Alfieri S (2020). Effects of sugammadex plus rocuronium vs neostigmine plus cisatracurium during renal transplantation on graft function: a retrospective, case-control study. Transplant Proc.

[CR41] Wagener G, Bezinover D, Wang C, Kroepfl E, Diaz G, Giordano C, West J, Kindscher JD, Moguilevitch M, Nicolau-Raducu R, Planinsic RM, Rosenfeld DM, Lindberg S, Schumann R, Pivalizza EG (2020). Fluid management during kidney transplantation: a consensus statement of the committee on transplant anesthesia of the American Society of Anesthesiologists. Transplantation..

[CR42] Younespour S, Rahimi Foroushani A, Maraghi E, Rostami Z, Einollahi B, Eshraghian MR, Mohammad K (2016). Longitudinal serum creatinine levels in relation to graft loss following renal transplantation: robust joint modeling of longitudinal measurements and survival time data. Nephrourol Mon.

